# Growth differentiation Factor 11 is an encephalic regionalizing factor in neural differentiated mouse embryonic stem cells

**DOI:** 10.1186/1756-0500-7-766

**Published:** 2014-10-29

**Authors:** Nele Vanbekbergen, Marijke Hendrickx, Luc Leyns

**Affiliations:** Department of Biology, Lab for Cell Genetics, Vrije Universiteit Brussel (VUB), 2 Pleinlaan, B-1050 Brussels, Belgium; The Belgian Scientific Institute for Public Health (WIV-ISP), Communicable and infectious diseases, Mycology and aerobiology, 14 J. Wytsmanstraat, B-1050 Brussels, Belgium

**Keywords:** Mouse ES cells, Brain patterning, GDF11

## Abstract

**Background:**

The central nervous system has a complex structural organization and consists of different subdomains along the antero-posterior axis. However, questions remain about the molecular mechanisms leading to the regionalization of this organ. We used a previously developed methodology to identify the novel patterning role of GDF11, a TGF-β signaling factor.

**Findings:**

Using an assay based on neural differentiated mouse embryonic stem cells, GDF11 is shown to induce diencephalic (posterior forebrain), mesencephalic (midbrain) and metencephalic (anterior hindbrain) fates at the expense of telencephalic (anterior forebrain) specification. GDF11 has not previously been implicated in the early patterning of the nervous system. In addition, inhibition of the TGF-β type I receptors Alk4, Alk5 and Alk7 by the pharmacological inhibitor SB431542 caused a strong anteriorization of the cells.

**Conclusions:**

Our findings suggest that GDF11 is involved in the earliest steps of the brain patterning during neurogenesis in the vertebrate embryo and is shown to be a regionalizing factor of the regional fate in the developing brain. This regionalization is not a typical posteriorizing signal as seen with retinoic acid, FGF or BMP molecules. To our knowledge, this is the first time that GDF11 is implicated in the earliest steps of the patterning of the neural plate.

## Introduction

Neural development is comprised of various processes that generate and form the nervous system during the earliest stages of embryogenesis. During early vertebrate development, the central nervous system (CNS) is subdivided along the antero-posterior (A/P) axis into forebrain, midbrain, hindbrain and spinal cord. Classical experiments in Amphibia suggest the existence of a two-step mechanism for this early organization. The neurectodermal tissue that is formed during the process of neural induction is initially anterior in character. It becomes secondarily posteriorized by a series of posteriorizing, or ‘transforming’ factors to obtain the full range of regional subtypes of the CNS along the A/P axis [1–3]. Several posteriorizing factors have been identified in amphibian embryos, such as Fgfs and Wnts [4–8]. Nevertheless, how the regionalization of the neural plate occurs in early mouse embryogenesis remains elusive.

An important tool to study embryonic development is mouse embryonic stem cells (mESc). mESc are derived from the inner cell mass (ICM) of a pre-implantation blastocyst stage embryo and have the capacity to self-renew unlimitedly *in vitro* in an undifferentiated state. Furthermore, they can be differentiated *in vitro* and *in vivo* into all cell types of the adult body [9, 10]. The parallelism between the differentiating embryo and the *in vitro* differentiation of mESc makes them an important tool to study embryonic development.

In a previous study [11], we developed a methodology to study mammalian early neural patterning which is based on the neural differentiation method of mESc as described by Ying and colleagues [12]. It involves the neural differentiation of mESc in the specialized serum-free N2B27 medium system in adherent cultures to obtain neural precursor cells. Subsequently the neural precursors were treated with potential posteriorizing factors [11, 12]. However, because many of the putative patterning factors (e.g. Bmp4, Wnt3a) were inhibitory to neural induction and some even had an effect on mESc self-renewal [13–18], we designed an experimental set-up that separated the neural induction from the neural patterning step, in order to avoid these negative effects on neural differentiation.

The signalling by the Transforming Growth Factor β (TGF-β) superfamily signalling is essential during a diverse set of cellular processes, including differentiation, patterning, proliferation, specification of developmental fate during embryogenesis as well as in mature tissue [19–21]. Members of the TGF-β superfamily include activins, inhibins, Bone Morphogenic Proteins (BMPs) and Growth of Differentiation Factors (GDFs). TGF-β factors initiate signalling by binding a heterodimeric complex of serine/threonine kinase transmembrane receptors, type I and type II [19–21]. The ligand first binds to the extracellular domain and activates a type II receptor homodimer, resulting in phosphorylation of a type I receptor homodimer. Once activated, the type I receptor directly phosphorylates and activates downstream a set of Smad proteins and initiates the intracellular signalling cascade. Type II receptors include BMPRII, ActRIIA, ActRIIB and T-β-RII. Type I receptors include seven members, activin-like kinases (ALK 1–7) [20, 22]. There are eight distinct Smad proteins: the receptor-regulated Smads, which include Smad1, 2, 3, 5 and 8; the Co-mediator Smad, Smad4 and the inhibitory Smads, which include Smad6 and 7 [19].

One of the members of the TGF-β superfamily, Growth of Differentiation Factor 11 (GDF11), also known as BMP11, has been shown to regulate anterior-posterior patterning of the body axis, kidney development and closure of the palate [23–27].

In the animal cap assay (AC) in *Xenopus*, GDF11 induces axial mesoderm and at higher concentrations also neural tissue, an activity that can be inhibited by Follistatin (Fst), indicating that in the AC assay, GDF11 has an effect similar to that of Activin [25]. In the mouse, GDF11 has been implicated in the establishment of the skeletal pattern. Mice that are mutant for this gene die within 24 hours after birth. They show homeotic anterior transformations of the vertebrae, mainly in the lumbar and the thoracic regions and a posterior displacement of the hindlimbs [27]. GDF11 regulates the patterning of the vertebrae by controlling the expression of the *Hox* genes, as the expression domain of several *Hox* genes is shifted in the mutants. In the chicken, it was shown that GDF11 not only causes a shift in the expression of *Hox* genes, but also causes a rostral shift in the position of the motor neuron columns and pools [28]. However, in the mouse embryo, it is not clear whether GDF11 has a patterning effect on other tissues than skeletal ones. In the mouse embryo, *GDF11* is expressed first faintly in the posterior half of the 7.5 dpc embryo where expression is observed in the primitive streak in the ingressing cells forming the mesoderm. At about 8.5 dpc, *GDF11* is expressed posteriorly; in the most anterior regions of the neural epithelium, and in both the neural epithelium and the mesoderm in more posterior regions. At 9.0 dpc, *GDF11* continues to be expressed in the former primitive streak region, and by 9.5 dpc, the expression is restricted mainly to the tail bud, but is also found in the posterior dorsal neural tube [27, 29]. It was reported that *GDF11* mRNA can also be detected in the encephalic region of 9.5 dpc and 10.5 dpc embryos [30]. These findings are consistent with a more general role of GDF11 during neural differentiation and expression in diverse neural tissues, which include developing spinal cord, dorsal root ganglia and embryonic and postnatal brain.

Based on this expression data and its skeletal patterning role, we hypothesized that GDF11 was a potential patterning factor that could be involved in the early neural A/P patterning of the mouse embryo. Therefore, in this study, we investigated whether GDF11 has a direct role in the early regional identity of neural progenitor cells and whether this factor can posteriorize freshly induced neural progenitors that are initially anterior in character. The potential neural patterning effect of GDF11 was assessed in our ES cell based patterning system. Our data suggest GDF11 is an encephalic regionalizing factor during early neural patterning in the vertebrate embryo.

## Research methods

### Mouse ESC cultures and differentiation

Mouse E14Tg2a feeder free ES cells were cultured in Knock Out DMEM (Gibco, Carlsbad, CA) supplemented with 10% fetal calf serum (Hyclone, Logan, UT), 0.1 mM β-mercaptoethanol (GIBCO), 1% non-essential amino acids (GIBCO), 2 mM L-glutamine (Sigma, St. Louis, MO), antibiotics (Sigma) and 1000 U/ml LIF (Sigma), on dishes coated with 0.1% gelatin.

For the patterning experiments, cells were cultured for 48 hours in N2B27 as described by Ying et al. [18] at a density of 7500 cells per cm^2^. Subsequently, the medium was replaced with N2B27 containing human recombinant GDF11 (R&D systems, Minneapolis, MN) and the TGF-β type I receptor inhibitor SB-431542 (Sigma). Culture occurred for another 4 days, refreshing the medium every 2 days.

To confirm the activity of the tested growth factors, cells were cultured similarly for 48 hours and collected 3 hours after treatment with the specific factor, and expression levels of known target genes were assessed. Three independent experiments were performed.

### Quantitative reverse transcription (qRT-PCR)

RNA was extracted from the collected cell samples using the SV Total RNA Isolation System kit (Promega, Madison, WI) and qRT-PCR was performed according to the method of Willems et al. [31].

Primer sequences for all markers used are available in the Real-Time Primer Database (http://medgen.ugent.be/rtprimerdb/). Expression levels were calculated as described using *Actb* as a reference gene [31]. In addition, expression levels of all regional marker genes were divided by the expression level of the panneural marker *Nestin* to correct for possible effects on neural differentiation [11].

Normalized expression levels of the treated samples were then calculated relatively to the expression levels of the corresponding untreated control, which was put at level one. The results are presented as the means of three independent experiments with the standard error of the mean. Statistical analysis was performed using a non-parametric test for comparing two groups (Mann Whitney). Statistically significant changes (p < 0.05) are marked with an asterisk in the graphs, as compared to control (sample 6).

## Findings

We developed a system to study early neural patterning and showed that Fgf2, Wnt3a and Bmp4 have a strong posteriorizing effect on neural differentiated mESc, similar to what was also described to be the case in *Xenopus* [1, 2, 11].

**Figure 1:**
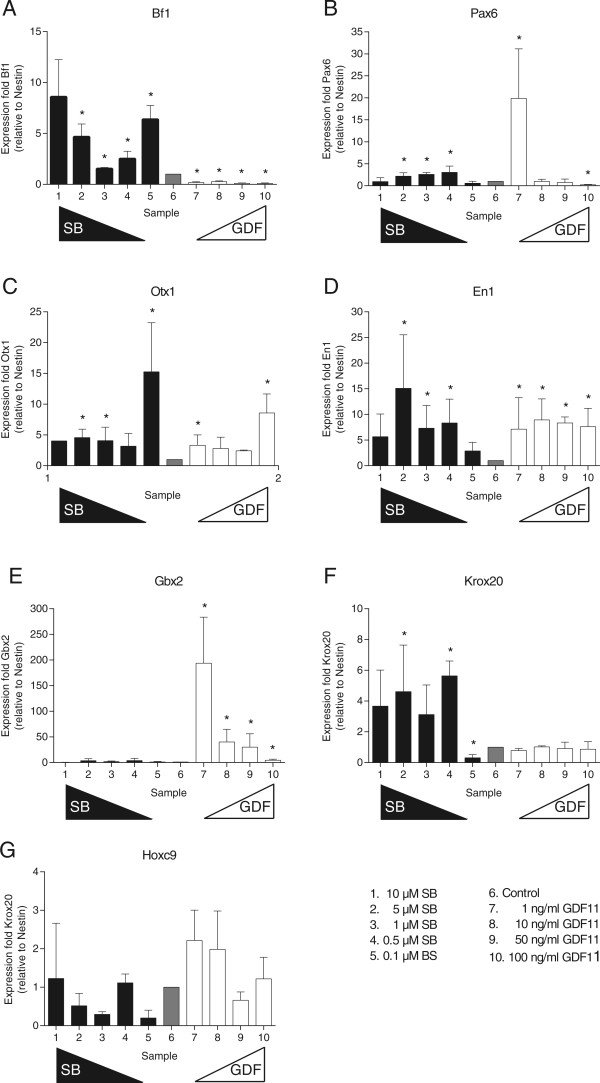
**Effect of SB431542 (SB), and GDF11 on neural patterning.** The effect of different concentrations of SB, and GDF11 on the expression level of different regional markers was assessed by qRT-PCR. The markers used were *Bf1* (telencephalon) **(A)**, *Pax6* (diencephalon) **(B)**, *Otx1* (prosencephalon and mesencephalon) **(C)**, *En1* (mesen- and metencephalon) **(D)**, *Gbx2* (metencephalon) **(E)**, *Krox20* (myelencephalon) **(F)** and *Hoxc9* (spinal cord) **(G)**. The results presented here are the means of three independent experiments with the standard error of the mean. Statistically significant changes in expression level (p < 0.05) are marked with an asterisk in the graphs, as compared to “control” (sample 6).

Analyses of the patterning potential of GDF11 showed that the addition of this factor induces a strong decrease of the anteriormost marker *Bf1*, and a significant upregulation of the intermediate markers *Otx1*, *En1* and *Gbx2* (Figure 1, panel A,C-E). On the posteriormost markers *Krox20* and *Hoxc9*, no effect was seen (Figure 1, panel F-G). The effect on the diencephalic marker *Pax6* was not unambiguous since at the lowest concentration tested, an increase was observed, while at the highest concentration tested, a decrease of *Pax6* expression was induced (Figure 1, panel B). Next, *Pax6* is expressed in neuroprogenitors in the developing spinal cord and not an exclusively in the diencephalon. This may contribute to the ambiguous results.

Treatment of the cells with SB431542 on the other hand, caused a significant upregulation of anterior markers like *Bf1* and *Otx1*, while at most of the concentrations tested, a downregulation of *Hoxc9* expression could be observed (Figure 1, panel A, C and G). This anteriorization indicates that endogenous posteriorization factors are actively signalling through the receptors blocked by SB431542.

GDF11 could indeed contribute to the endogenous patterning of the cells that could be inhibited by SB431542, since its expression, as well as the expression of the Alk4 and Alk5 type I receptors could be detected in the control cultures by qRT-PCR (data not shown).

**Figure 2:**
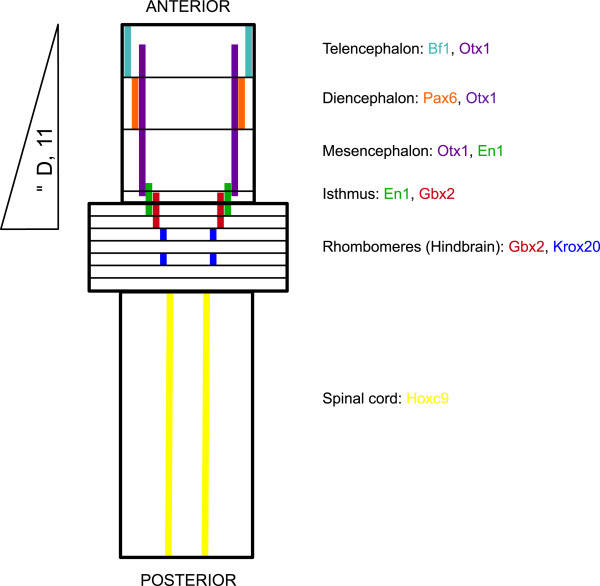
**Schematic diagram of the probable effect of GDF11 on the marker genes used and their respective expression patterns.** Anterior is towards the top (adapted from Reichert H, 2002 [32]). Expression domains are colour coded: the expression domain of Bf1 is shown in the colour light blue, Otx1 domain in purple, Pax6 domain in orange, En1 domain in green, Gbx2 domain in red, Krox20 domain in dark blue and Hoxc9 in yellow, respectively.

In all gene expression data tested above, the expression levels of all regional markers (see Figure 2 for schematic diagram) were put relative to the expression level of the panneural marker *Nestin* in order to correct for possible inhibitory effects of the patterning factors tested on neural induction. Though in our experimental set-up, the neural induction process was separated in time from the neural patterning process, an inhibitory effect on *Nestin* expression could be seen by GDF11 treatment. Flow cytometric analysis was used to assess the percentage of Nestin-positive cells after treatment with these factors. Treatment of the cells with the highest concentrations of GDF11 reduced the number of Nestin-positive cells by 20% respectively. This indicates that in our experimental set up the inhibitory effect of GDF11 is limited, but could not completely be avoided. Therefore we corrected all expression data for the expression level of Nestin. These data indicate that GDF11 exerts a fundamental role during neural development.

## Discussion

Though a neural patterning role is well established for Activin in *Xenopus* and in Zebrafish [33, 34], we did not detect any posteriorizing effect by recombinant Activin A in our mES cell based patterning system (data not shown). Nodal, well known for its crucial role in the establishment of the A/P body axis, was the next candidate we tested. More precisely, the Nodal antagonists *Lefty1* and *Cerberus-like* that are expressed in the anterior visceral endoderm (AVE) of the mouse embryo, are essential for the anterior neural specification [35, 36]. Whether Nodal itself can posteriorize the neural tissue in the mouse has not directly been shown and addition of recombinant Nodal to the neural precursor cells in our system did not directly induce any posteriorization either (data not shown).

Since SB431542 was tested and shown to affect A/P patterning of the brain, we searched for other candidates like Nodal or Activin that could signal via the Alk systems blocked by SB431542.

Several GDFs (GDF1, GDF3, GDF8, GDF9 and GDF11) were shown to signal through the Alk4, Alk5 or the Alk7 receptor [22, 37–40]. GDF11 was shown to signal preferentially through the Alk5 receptor and has been implicated in the establishment of the skeletal pattern [22, 27]. Whether this factor also has an effect on the regionalization of the mouse neural tube remained elusive. In chick however, overexpression of *GDF11* in the neural tube caused a rostral displacement of the *Hox* expression domains and the motor neuron columns [28]. During the present study, we investigated whether GDF11 has a direct role in the early regional identity of neural progenitor cells and whether this factor can posteriorize freshly induced neural progenitors that are initially anterior in character. The potential neural patterning effect of GDF11 was assessed in our ES cell based patterning system.

GDF11 was indeed shown to be a strong patterning factor, reducing the expression levels of the most anterior markers *Bf1* (ventral telencephalon) and upregulating the expression of more posterior neural markers like *En1* (isthmus, midbrain to midbrain-hindbrain junction) and *Gbx2* (hindbrain). The most posterior marker, *Hoxc9* (neural tube), tested remained unaffected, indicating that the role of GDF11 is limited to the developing brain. More specifically, GDF11 induces midbrain and anterior hindbrain fates at the expense of telencephalic fates. This is the first time that this factor is implicated in the regionalization of the brain during early mouse development, where GDF11 could play a more permissive or maintenance role for the expansion of certain progenitors (i.e. Otx1+, En1+ progenitors). Furthermore, the presence of *GDF11, Alk4* and *Alk5* mRNAs in the differentiated ES cells indicates that GDF11 may be endogenously signalling in our system. However, the observed effect for GDF11 was not completely opposite to the one observed by adding SB431542, for example both GDF11 and SB431542 caused a significant upregulation of anterior marker *Otx1* (forebrain, but not most rostral part, and midbrain), as compared to the opposite effect observed on *Bf1*. SB431542 might exert a pleiotophic effect, inhibiting Alk4, Alk5 as well as Alk7 [41, 42]. This suggests that other TGF-β factors signalling through these receptors (including other GDFs), may have an additional patterning function in the developing neural tube and that SB431542 inhibits all of these effects. This still needs further investigation.

Because of this clear regionalizing effect of GDF11, we also tested Follistatin, a GDF11 antagonist, for its patterning potential, but no significant changes in the expression levels of the regional markers could be observed (data not shown). It might be possible that, blocking receptors by pharmalogical inhibitors, such as SB431542, is more efficient than the addition of proteins antagonizing the signalling factor because of the long experimental culture period.

At the developmental stage where the early regionalization of the neural tube and encephalic region is established, *GDF11* was predominantly expressed in the posterior spinal cord. This expression pattern was not fully compatible with the observed encephalic regionalization effect, since this was restricted anteriorly to the level of the hindbrain. However, *GDF11* expression was also detected in the brain region by using the radioactive *in situ* hybridization technique [30]. Our results suggest a localized activity of GDF11 in the metencephalic region of the brain but how this regional activity in the posterior encephalic region of GDF11 is established, remains unclear. Future research is needed to determine if GDF11 is acting together with another signalling molecule, possibly another member of the TGF-β superfamily or if localized, possibly graded, GDF11 signalling inhibitors are involved in the patterning effect observed. Finally, it should be noted that the regionalization by GDF11 is not a typical posteriorizing signal as seen with retinoic acid, FGF or BMP molecules since these also lead to an increase of more posterior markers while GDF11 is an encephalic regionalizing factor.
